# Enhancing Omics Analyses Through Coalitional Games and Shapley Values

**DOI:** 10.3390/mps9010025

**Published:** 2026-02-12

**Authors:** Eva Vargas, Inés de la Torre, Francisco J. Esteban

**Affiliations:** Systems Biology Unit, Department of Experimental Biology, Faculty of Experimental Sciences, University of Jaén, 23071 Jaén, Spain

**Keywords:** coalitional games, game theory, omics data, Shapley values, systems biology

## Abstract

We describe a comprehensive methodology for the application of game theory to omics data analysis, with a particular focus on coalitional games and Shapley values. This approach evaluates the cooperative distribution of genes within high-dimensional transcriptomics datasets, providing a complementary perspective to conventional statistical methods. We present the mathematical framework, implementation details, and references for applications that demonstrate its ability to improve the detection of biologically meaningful signals that may not be explicitly modeled by many conventional statistical methods. Our results highlight the potential of coalitional game theory as a powerful tool for enhancing reproducibility and interpretability in omics research, opening new perspectives in systems biology and precision medicine.

## 1. Introduction

The challenging analysis of high-dimensional omics datasets, particularly at the transcriptomic level, represents a milestone in systems biology. High dimensionality, technical noise, and variability between analysis pipelines hinder the detection of biologically relevant signals and reduce reproducibility between studies [1,2]. Traditional statistical approaches based on independent gene contrasts, *p*-values, and multiple testing corrections lose power in these scenarios and, moreover, fail to capture the cooperative relationships between genes that underlie complex biological processes [3]. Even more sophisticated frameworks, such as differential expression analysis or network-based methods, often struggle to model these interdependencies [4]. Despite recent advances in statistical modeling and machine learning, many conventional approaches still face substantial methodological limitations when dealing with the inherent complexity of gene interactions [1,5].

When two entities (organisms, individuals, systems) interact, the outcome could vary widely depending on the intention of each party involved. These interactions can be quantitatively examined using game theory models [6]. Game theory, particularly the so-called branch of coalitional games, provides a mathematically principled framework to study cooperation and contribution among interacting entities [1,7,8]. When applied to gene expression data, each gene can be conceptualized as a “player” in a cooperative game, where the value function quantifies the collective performance of subsets of genes [9]. The Shapley value—a fundamental concept in cooperative game theory—offers a fair and interpretable way to distribute this collective value among individual genes, reflecting their relative importance within biological processes [8]. Applied to omics data, this concept allows us to quantify the relevance of each gene by taking into account not only its individual behavior but also its participation within multiple expression configurations associated with a given phenotype.

Here, we describe a comprehensive and reproducible methodology for applying coalitional game theory and Shapley values to omics data. We detail the mathematical background, implementation strategies, and validation through case studies on hypothetical transcriptomics datasets. This protocol is intended to serve as a practical reference for computational biologists seeking to integrate cooperative models into high-dimensional data analysis. Finally, we discuss and summarize some of the main findings regarding the applicability of game theory-based methods to the research on biomarker discovery. This integration of game theory and omics analysis opens new perspectives for systems biology and precision medicine.

## 2. Mathematical Workflow

In this section, we formalize the cooperative game theory-based method for evaluating the joint relevance of genes in transcriptomic data. The approach is built upon the definition of coalitional games, Boolean encoding of expression properties, and value assignment using the Shapley value, and it has been described in detail previously [1,7,10]. The goal is to quantify how the cooperative contribution of each gene varies between two experimental conditions (e.g., controls vs. cases).

### 2.1. Data Set Representation

Consider a normalized and preprocessed transcriptomic dataset consisting of:

A set of genes $G=\left\{g_{1},g_{2},\ldots ,\;g_{n}\right\}$,

A set of samples $S=\;S_{C}\cup S_{E}$, where

$S_{C}$ represents control samples and $S_{E}$ represents cases.

For each gene $g_{i}$ and sample $s_{j}$, the normalized expression value $X_{ij}$ is available.

### 2.2. Boolean Coding of Expression Properties

To define gene coalitions, each expression value is converted into a binary variable indicating whether the gene exhibits a property of interest in the sample. Two complementary properties are considered: overexpression and underexpression.

Let $\mu_{i}^{C}$ and $\sigma_{i}^{C}$ be the mean and the standard deviation of the gene $g_{i}$ in the control group, respectively. We define two indicator functions:

(a) Overexpression matrix $B^{+}\left\{\begin{array}{c} \;1,\;if\;X_{ij}\geq \;\mu_{i}^{C}+\;\sigma_{i}^{C}\;\\ \;0,\;otherwise \end{array}\right.$
(b) Underexpression matrix $B^{-\;}\left\{\begin{array}{c} \;1,\;if\;X_{ij}\leq \;\mu_{i}^{C}-\;\sigma_{i}^{C}\;\\ \;0,\;otherwise \end{array}\right.$

These matrices collect, for each sample, the discretized properties that will constitute the coalitions of the game.

### 2.3. Sample-Defined Coalitions

A column of $B^{+}$ or $B^{-}$ represents a sample $s_{j}$.

Let $C_{j\;}\subseteq G$ the set of genes with a value of 1 in the sample:

$$C_{j}=\;\left\{g_{i}\;\in G:\;B_{ij}^{\pm }=1\right\}$$

This set is interpreted as a winning coalition: genes that jointly manifest the property of expression in that sample.

Notation:

For controls: coalitions $C_{j}^{C}.$

For cases/experimental groups: coalitions $C_{j}^{E}.$

### 2.4. Definition of the Coalition Game

Let N=G be the set of players (genes). A cooperative game is defined as a pair (N, v), where

$$v:\;2^{N}\to R$$

is a utility function that assigns a value to each coalition.

In microarray games, the function v is defined to reflect sufficiency: an observed coalition receives a value of 1, and unobserved coalitions receive 0. For each sample $s_{j}$:

$$v\left(C_{j}\right)=1,\;v\left(S\right)=0\;if\;S\neq C_{j}.$$

For the sample set of a condition, utility is extended by summing the player’s marginal contribution to each observed coalition.

### 2.5. Marginal Contribution

Given a gene $g_{i}$ and a coalition $C_{j}$ of size $\left|C_{j}\right|$, the marginal contribution of the gene is defined as:

$$m\left(g_{i},C_{j}\right)=\;\left\{\begin{array}{cc} \frac{1}{\left|C_{j}\right|}, & \;if\;g_{i}\in C_{j},\\ 0, & \;if\;g_{i}\;\notin \;C_{j}. \end{array}\right.$$

This definition assumes an equal distribution of utility among the genes that make up the winning coalition.

### 2.6. Shapley Value

The Shapley value is a classic solution in cooperative games that measures a player’s average contribution across all possible coalitions. In this context, the Shapley value of the gene $g_{i}$ in a condition (control or experimental) is calculated as the average of its marginal contributions across all samples of that condition:

$$\phi_{i}=\;\frac{1}{\left|S\right|}\;\sum_{j\in S}m\left(g_{i},C_{j}\right).$$

Thus, we obtain $\phi_{i}^{C}$ as the Shapley value in controls, and $\phi_{i}^{E}$ as the Shapley value in experimental subjects or samples. Regarding its interpretation, $\phi_{i}$ measures how cooperatively relevant a gene is within the coalitions associated with the given condition.

### 2.7. Absolute Difference in Shapley Values (ADSVs)

The key measure for identifying differentially relevant genes between conditions is:

$${ADSV}_{i}=\;\left|\phi_{i}^{C}-\phi_{i}^{E}\right|$$

A high value indicates that the cooperative contribution of the gene changes substantially between conditions, even if the differences in means or traditional *p*-values do not detect it.

### 2.8. Bootstrap Significance Estimation: CASh

To distinguish between real changes in cooperative contributions and random fluctuations, a resampling procedure is applied:

1. The samples are randomly re-labeled as control and experimental.
2. The following values are recalculated, $\phi_{i}^{C*}$, $\phi_{i}^{E*}$, and their difference ${ADSV}_{i}^{*}$.
3. The process is repeated *B* times (typically 1000).
4. The non-parametric *p*-value is defined as:

$$p_{i}=\frac{1}{B}\;\sum_{b=1}^{B}I({ADSV}_{i}^{*}\geq {ADSV}_{i})$$

where $I\left(\cdot \right)$ denotes an indicator function that equals 1 when the condition is satisfied and 0 otherwise.

This procedure is called CASh (Comparative Analysis of Shapley values).

Genes are considered candidates if they simultaneously fulfill:

- $p_{i}\leq \alpha$ (although bootstrap-based *p*-values can increase sensitivity, standard multiple-testing correction procedures, such as false discovery rate control, remain necessary in high-dimensional omics analyses to limit false-positive findings),
- ${ADSV}_{i}\geq \mu_{ADSV}+\sigma_{ADSV}$ in its group.

The increased power of the CASh procedure arises from its ability to stabilize Shapley value estimates through repeated resampling while preserving the cooperative structure of the data. By evaluating marginal gene contributions across multiple bootstrap replicates, CASh mitigates the impact of sample-specific noise and outliers, which are common in omics datasets. This aggregation across resampled coalitions enhances sensitivity to consistent but subtle effects that may not reach significance under classical differential testing, particularly in heterogeneous biological conditions.

### 2.9. Final Gene Selection

The final set of relevant genes is defined as:

$$G^{\backslash *}=\left\{g_{i}\in G:\;p_{i}\leq \alpha \;and\;{ASDV}_{i}\geq \mu +\sigma \right\}$$

This set represents genes whose differential cooperative relevance is associated with the experimental condition.

## 3. Implementation

The first step is to build a Boolean matrix including all the arrays of a specific condition where the Boolean values 0–1 represent two complementary expression properties, for example, the normal expression (coded by 0) and the overexpression (coded by 1). From a practical point of view, and avoiding a formal mathematical description, for a given array included in a Boolean matrix, the *support* of that array is considered as the group of genes coded by 1 (the winning coalition on that single array); if seven genes were coded by 1, we may say that the array has a support of seven, and the marginal contribution of any gene belonging to this coalition is 1/7 (because we coded all these genes by 1 and we consider all of these genes to have the same quantitative participation in the disease). The microarray game corresponding to this matrix is then defined as a coalitional game where a well-known solution is the Shapley value, which assigns to each player (gene) their average marginal contribution. For example, if the same gene is coded by 1 in a different coalition with a support of eight, its marginal contribution in this second coalition is 1/8, and let us suppose that five arrays (five patients) were analyzed; then, 0.0536 is the average marginal contribution (Shapley value) of this gene (i.e., (1/7 + 1/8)/5).

For a more descriptive point of view, let us use as an example a final matrix (Table 1) including the expression levels of nine genes and ten samples (five controls, C, and five experimental cases, E). The following toy example is intentionally designed for illustrative purposes, with the sole aim of clarifying the mechanics of the proposed workflow. It is not intended as a performance benchmark, but rather as a didactic demonstration of how coalitional games and Shapley values are constructed and interpreted in an omics context.

First, in order to discriminate over-regulated levels of gene expression with respect to expression measured in controls, each continuous value in the vector $X_{i}=(X_{i1},\;\ldots ,\;X_{i10})$ which is equal to or greater than $Mean\left[X_{i.}^{C}\right]+Stdev[X_{i.}^{C}]$ is coded as 1 and 0 otherwise. Consequently, a Boolean matrix $B^{+}$ with nine rows and ten columns and with values {0, 1} is generated from X (Table 2).

Separately, a procedure aimed to discriminate under-regulated levels of gene expression with respect to expressions measured also in controls is applied. Each continuous value in the vector $X_{i.}={(X}_{i1},\ldots ,\;X_{i10})$, which is equal to or smaller than $Mean\left[X_{i.}^{C}\right]-Stdev\left[X_{i.}^{C}\right]$, is coded as 1 and 0 otherwise. Consequently, a Boolean matrix $B^{-}$ with 9 rows and 10 columns with values {0, 1} was also generated from X (Table 3).

According to the distinction between control and experimental samples, the Boolean matrix $B^{+}$ is split into two different matrices $B^{C+}$ and $B^{E+}$, and the Boolean matrix $B^{-}$ is split into two other different matrices $B^{C-}$ and $B^{E-}$. Then, from the Boolean matrix $B^{E+}$, the corresponding Shapley values are calculated (Table 4). In a similar way, the corresponding Shapley values from the Boolean matrix $B^{E-}$ are also obtained (Table 5).

In order to remove genes with high Shapley values that could be attributed to chance, Moretti et al. (2008) introduced a bootstrap resampling technique procedure over the observed Shapley values (Comparative Analysis of Shapley value; shortly, CASh); moreover, a stimulation study suggested that CASh offers more power than *t*-test for the detection of differential gene expression variability [1].

Finally, and as an additional filtration step in each microarray game, genes showing both an absolute difference in the Shapley value (ADSV; for any given gene, the Shapley value obtained from the $B^{C+}$ matrix (Table 6) minus its corresponding value obtained from the matrix $B^{E+}$ (Table 4); or the Shapley value obtained from the $B^{C-}$ matrix (Table 7) minus its corresponding value obtained from the matrix $B^{E-}$ (Table 5); ADSV column in Table 8 and Table 9) greater than the mean plus the standard deviation for each group and a *p*-value ≤ 0.05 from the bootstrap re-sampling (CASh column in Table 8 and Table 9), can be selected as candidate genes for further validation or functional analysis. In the given example, genes 7 and 8 showed both criteria and, thus, they can be considered as differentially regulated in a microarray experiment (Table 8 and Table 9).

In this example, the application of the selection criteria results in a list of potential targets for further analyses. Specifically, gene7 and gene8 emerge as candidates for downstream validation, as they satisfy the requirements of statistical significance in the bootstrap resampling procedure (CASh column in Table 8 and Table 9) and a Shapley value difference (ADSV column in Table 8 and Table 9) exceeding the established threshold for their respective groups. Therefore, in this context, these two genes represent plausible biologically relevant signals and warrant further exploration through functional annotation or experimental follow-up.

Thus, the implementation of the microarray game follows a structured sequence of operations. First, gene-expression values are transformed into Boolean matrices according to the selected thresholding criterion, generating separate representations for over- and underexpression in cases and controls. Each Boolean matrix is then interpreted as a cooperative game, where genes act as players and samples define coalitions. Shapley values are computed for each game to quantify the marginal contribution of individual genes. Finally, the CASh procedure is applied to these values to assess statistical significance through bootstrap resampling, enabling the identification of genes whose cooperative contributions are robust across resampled datasets.

### Computational Considerations and Scalability

The computational cost of the proposed workflow is primarily driven by the estimation of Shapley values and the number of bootstrap resamples used in CASh. While exact Shapley value computation is exponential in the number of features, the present framework is intended to operate on reduced feature sets obtained after preliminary filtering. Accordingly, the application of coalitional game theory and Shapley value estimation to omics data implicitly assumes a reduced and informative feature space [1,10]. In high-dimensional transcriptomic settings, operating directly at the genome-wide scale is neither computationally tractable nor conceptually optimal, as the inclusion of a large number of weakly informative or noisy features dilutes cooperative effects and obscures meaningful marginal contributions [2,3].

For this reason, a preliminary feature preselection step is considered appropriate and necessary to define a plausible set of players for the cooperative game [10,11,12]. Importantly, this preselection does not constitute a final criterion for biological relevance or statistical significance. Instead, it serves as a dimensionality-reduction strategy that preserves genes exhibiting minimal individual signal, while deferring all decisions regarding cooperative relevance and statistical robustness to the Shapley value differences and the CASh resampling procedure [10,11,12]. In this sense, preselection defines the space of interaction, whereas inference is entirely driven by the cooperative analysis.

In practice, CASh is best suited as a prioritization or refinement step rather than a genome-wide inference tool. Reasonable bootstrap sizes (typically on the order of 1000 resampling iterations, applied to reduced microarray matrices (including the preselected genes) provide stable estimates while maintaining tractability, allowing the method to be applied to real transcriptomic datasets as demonstrated in previous studies [10,11,12].

## 4. Discussion

Cooperative game theory, along with Shapley value calculations, has become an increasingly important tool in omics data analysis. Its ability to quantify the individual contribution of genes, variants, or genomic regions within highly interdependent contexts makes it a valuable complement to traditional statistical methods.

A key component of the proposed workflow is the Boolean encoding of gene-expression values, which enables the formulation of cooperative games from transcriptomic data. This transformation represents a deliberate simplification aimed at highlighting coordinated expression patterns rather than precise quantitative changes. Nevertheless, real omics datasets—particularly RNA-seq and single-cell data—often exhibit skewed, heavy-tailed, or zero-inflated distributions. Under such conditions, the choice of thresholding strategy may influence the resulting Boolean representation and, consequently, the inferred cooperative structure. While the framework remains applicable in principle, alternative encoding schemes or preprocessing steps may be required when gene-expression distributions deviate substantially from the assumptions implicit in microarray data.

Some of the main advances achieved through cooperative game theory, along with Shapley value calculation approaches, are summarized below.

### 4.1. Microarray Data Analysis

The use of game theory-based methods in transcriptomics formally began with the pioneering work of Albino et al. (2008), who applied a game theory approach to evaluate microarray data for the first time [7]. By combining the Significance Analysis of Microarrays (SAM) and cooperative analysis, they identified genes with low intratumoral heterogeneity in neuroblastic tumors, demonstrating that these methods can highlight biological signals that are difficult to detect with conventional techniques [7].

Along similar lines, Moretti et al. (2010) showed that the Shapley value allows for quantifying the “power” or influence of each gene interacting with others, particularly highlighting the role of hub genes in functional networks [8]. This work introduced improvements in the resolution of gene interactions through an analysis based on pairwise relationships [8].

Regarding contributions from our group, Esteban & Wall (2011) demonstrated that combining the microarray game and CASh approaches substantially increases the power to detect disease-related genes in microarray datasets with limited differential-expression signal [10]. Their analysis highlighted how cooperative game theory approaches can uncover biologically meaningful genes that remain hidden under conventional statistical frameworks, particularly in conditions characterized by high heterogeneity. More recently, Castro-Martínez et al. (2024) applied CASh to the analysis of multiple uterine pathologies, including endometriosis, endometrial cancer, and leiomyomas [11]. The method improved robustness against outliers and allowed the detection of molecular signatures associated with these diseases, highlighting its usefulness for refining transcriptomic analyses in complex clinical contexts [11].

### 4.2. Co-Expression Networks and Gene Prioritization

Game theory has proven particularly effective for studying complex gene networks [8]. Cesari et al. (2018) used the Shapley value to determine the relevance of each gene within a co-expression network, highlighting the functional importance of peripheral genes in the regulation of biological pathways [13]. Furthermore, they compared this approach with classical centrality indices and proposed its integration with clustering techniques to improve the identification of relevant genes [13].

Additional work has reinforced this line of research, showing that Shapley values can serve as more sensitive and functionally interpretable centrality metrics than conventional measures [14].

### 4.3. Applications to Complex Diseases: Autism as a Case Study

Autism Spectrum Disorder (ASD) has become one of the main fields where large-scale cooperative methods have been implemented.

Gupta et al. (2018) applied coalitional game theory to 1965 fully sequenced genomes from 756 multiplex families [9]. By encoding disruptive gene mutations in binary arrays for cases and controls, they calculated Shapley values for each gene, identifying 67 genes with significantly high contributions to the ASD phenotype. These genes corresponded to biological pathways previously linked to the disorder, demonstrating that coalitional game theory can reveal “hidden players” in polygenic diseases [9].

Following this line of research, Sun et al. (2019) extended the analysis to non-coding regions, applying Shapley values to 4595 fully sequenced genomes [15]. They identified 30 non-coding positions having significantly elevated player scores, likely representing significant contributors to the genetic environment underlying ASD. This work highlights how Shapley values allow the detection of synergistic contributions underpinning complex neurodevelopmental disorders such as autism [15].

Subsequently, Sun et al. (2020) incorporated prior knowledge using a Shapley-based centrality metric applied to gene interaction networks [14]. This approach allowed for the prioritization of genes with synergistic influence on network connectivity, highlighting HLA genes involved in immunity and previously associated with ASD. The study demonstrated the usefulness of combining game theory and networks to decipher polygenic associations to complex disorders [14].

In addition to these large-scale genomic studies, work from our group has shown that cooperative approaches can also enhance the analysis of transcriptomic data in ASD. In their study, Esteban & Wall (2011) applied an approach combining microarray game and CASh to an ASD expression dataset, demonstrating that this framework can detect gene expression patterns that traditional tests overlook [10]. Rather than focusing on individual gene-level changes, their approach emphasized how groups of genes jointly contribute to disease-related expression profiles, revealing functionally coherent sets linked to neurological and immune processes [10]. More recently, we demonstrated CASh as a useful tool for the detection of differentially expressed genes in neuropsychiatric disorders such as autism, schizophrenia, bipolar disorder, and major depression, proving again this approach to be more sensitive than classical differential methods, especially in identifying subtle but biologically relevant patterns [12].

### 4.4. Integration with Protein–Protein Interaction Networks and Cancer Biomarkers

The field of cancer has also embraced game theory-based tools. Farahmand et al. (2016) proposed a game theory approach that integrates transcriptomic profiles and protein–protein interaction networks [16]. Applying it to three breast cancer datasets, they identified metastasis-marking subnetworks that outperformed biomarkers based individually on genes, pathways, or network topology. Furthermore, they revealed a novel candidate gene for breast cancer susceptibility [16].

Additionally, Farahmand et al. (2017) developed CytoGTA, a Cytoscape plugin that facilitates the identification of discriminative subnetwork markers between phenotypes using cooperative game theory, making the methodology widely accessible and simple to use to biologists and bioinformaticians [17].

### 4.5. Post-Transcriptional Regulation and miRNA-mRNA Synergies Modeled Using Game Theory

Game theory has also been applied to the field of post-transcriptional regulation. In this context, Serra et al. (2021) developed miRgame, a game theory-inspired model that quantifies the joint contribution of multiple miRNAs acting on each Ago2 peak in CLIP-seq data [18]. The model summarizes the cooperative occupation of miRNAs and allows for the stratification of binding sites according to their repressive potential. The authors observed that greater cooperative occupation is associated with more intense gene repression, showing that miRNAs can act synergistically to modulate the expression of their targets [18].

### 4.6. Hybrid Methods: Game Theory, Deep Learning and Multimodal Data

One of the most recent applications involves the use of Shapley values to interpret deep learning models. In a study on alternative splicing biomarkers associated with motor deficits due to prenatal alcohol exposure and maternal diabetes, Dutta et al. (2023) used Shapley values to determine which splicing events contributed most to model performance, identifying 29 common events with high predictive power [19].

Similarly, although not focused exclusively on transcriptomics, Pappas et al. (2020) showed how Shapley values can link structural and functional brain connectivity with specific gene expression patterns associated with neurotransmission [20].

The integration of deep learning with game theory-based interpretability methods continues to expand its presence in transcriptomic analysis. In a recent study on temporal lobe epilepsy, Wang et al. (2025) developed an interpretable diagnostic model from RNA-seq and microarray data, comparing various algorithms until they obtained a diagnostic neural network that achieved perfect performance [21]. To unravel the molecular determinants of the model, they used Shapley-Analog Potential (SAP), identifying key genes associated with temporal lobe epilepsy pathogenesis, and complemented the analysis with Kolmogorov–Arnold networks to capture additional nonlinear relationships. The work demonstrates how Shapley values can bring transparency to complex models and facilitate their integration into gene expression-based clinical applications [21].

The applicability of Shapley values as an interpretability tool extends to multi-omics studies focused on identifying clinically relevant biomarkers. In a comprehensive analysis of pancreatic cancer, Sun et al. (2025) integrated transcriptomic, proteomic, single-cell, and spatial transcriptomics data to characterize the role of the SH3-domain kinase binding protein 1 (SH3KBP1) [22]. Multiple machine learning algorithms converged on selecting this gene as a predictive marker, and the use of Shapley Additive Explanations (SHAP) confirmed its crucial contribution to model performance. Further validation revealed that SH3KBP1 is overrepresented in malignant epithelial cell populations and associated with a poorer prognosis. This study demonstrates how Shapley values facilitate the interpretation of complex models and strengthen the identification of robust biomarkers within integrated multi-omics strategies [22]. In addition, a one-shot learning method based on Siamese networks that integrates gene expression data and mutational profiles for cancer detection has been developed recently. Using SHAP, the authors were able to interpret the model’s decisions and highlight the genes and mutations with the greatest impact on classification, showing that combining transcriptomic and genomic information can improve biomarker identification in data-scarce contexts [23].

Taken together, these studies demonstrate how SHAP and other Shapley value-based methods have evolved into essential tools for interpreting predictive models, identifying biomarkers, and improving classical transcriptomic analyses. Their versatility allows for their application to multi-omics data, deep learning models, survival analysis, and differential expression studies, solidifying game theory as an analytical cornerstone of modern biomedical research.

### 4.7. Final Remarks

The body of evidence presented in this work illustrates how game theory approaches, and particularly Shapley value-derived metrics, have progressively evolved from exploratory tools in microarray studies into analytical frameworks applicable across a broad spectrum of biomedical conditions. Their capacity to quantify the marginal contribution of genes, variants, or molecular features within highly interdependent biological systems enables a level of interpretability that complements and often emphasizes coordinated contributions among features alongside statistical and machine learning methods. Importantly, Shapley-based strategies offer a principled way to assess nonlinear interactions and synergistic effects, which are frequently overlooked by traditional approaches such as DESeq2 [24] or simple feature-ranking methods, and provide more stable explanations than techniques like LIME [25] in high-dimensional omics settings.

A central practical aspect concerns the applicability of the proposed framework to high-dimensional, low-sample-size settings, which are typical of modern omics studies [26]. In such scenarios, the method is not intended to operate directly at the genome-wide scale, but rather as a structured filtering and prioritization layer applied after preliminary dimensionality reduction or feature preselection. The cooperative game formulation is particularly suited to contexts in which complex interactions exist among a reduced set of candidate features, allowing the identification of coordinated contributions that classical marginal statistics often fail to detect. An additional aspect of relevance for complex diseases is the ability of the CASh procedure to mitigate biological and technical heterogeneity. By aggregating Shapley value estimates across multiple bootstrap resamples, CASh reduces the influence of sample-specific fluctuations and emphasizes cooperative gene contributions that are consistently observed across resampled coalitional contexts. This resampling-based stabilization is particularly advantageous in heterogeneous disorders, where subgroup structure or inter-individual variability may obscure biologically meaningful signals under single-pass analyses. Importantly, this strategy does not replace explicit modeling of heterogeneity through hierarchical or mixed-effects approaches, but rather complements them by providing robust cooperative attribution in the presence of variability. In addition, the proposed cooperative game theoretic framework is not intended to replace existing high-dimensional inference or network-based variable selection methods. Instead, it should be viewed as complementary to established approaches, such as network-regularized regression models, which explicitly incorporate interaction structures and scale efficiently to large datasets [27]. Therefore, the present method offers a different perspective, focusing on the attribution of cooperative contributions among features, and may therefore be particularly useful as a refinement or prioritization step within broader analytical pipelines.

Despite these advantages, several challenges and limitations remain. In high-dimensional settings, Shapley value estimation and bootstrap-based significance assessment are computationally demanding, and exhaustive coalitional evaluation becomes infeasible without prior feature reduction. As dimensionality increases, both runtime and memory requirements scale rapidly, making the direct application of the framework to genome-wide datasets impractical. Consequently, the proposed method should not be interpreted as a stand-alone high-dimensional inference engine, but rather as a refinement and attribution tool operating on reduced feature spaces.

Additional limitations stem from methodological choices. The performance of Shapley-based analyses depends strongly on the definition of the value function and the underlying model architecture, while bootstrap resampling introduces a trade-off between stability and computational cost. In deep learning and multimodal frameworks, approximation strategies are often required to make Shapley estimation tractable, potentially affecting accuracy. Moreover, although Shapley values quantify feature contribution, they do not inherently capture causal relationships, underscoring the need for careful experimental validation.

Future research directions point towards increasingly integrative applications. Single-cell RNA-seq, for example, provides an ideal setting for cooperative game analysis: the heterogeneity of tissues could be better characterized through Shapley-based evaluations of cell-type-specific markers, gene–gene interactions, and regulatory circuits. Similarly, multi-omics frameworks combining transcriptomics, epigenomics, proteomics, and spatial information stand to benefit from cooperative models capable of mapping feature interactions across different molecular layers. At the network level, incorporating Shapley values into gene regulatory, protein interactions, or cell–cell communication networks could improve the detection of synergistic modules and potential candidate biomarkers.

Beyond transcriptomics, the proposed workflow could be naturally extended to multi-omics settings by defining players as genes, proteins, metabolites, or integrated molecular entities across layers. In complex disease contexts such as cancer therapeutics [28] or metabolic disorders [29], cooperative game-based indices may help disentangle synergistic cross-omics contributions and prioritize molecular components with system-level relevance. Recent advances in integrative omics analysis suggest that such cooperative formulations may provide an additional layer of interpretability in precision medicine applications [30,31].

Finally, and beyond methodological advances, these approaches hold promise for strengthening reproducibility and enhancing precision medicine. By providing transparent explanations of model predictions, Shapley-based methods facilitate clinicians and researchers to trace biomarker relevance back to underlying data, and support the development of interpretable diagnostic and prognostic tools. As biomedical datasets continue to expand in complexity and size, cooperative game theory is poised to become an indispensable analytical axis, bridging predictive performance with biological interpretability and bringing computational models closer to clinical translation.

## 5. Conclusions

In this work, we present a comprehensive methodological framework for applying cooperative game theory to the analysis of omics data, with a particular emphasis on the use of coalition games and Shapley values to transcriptomics data. This approach allows for the rigorous evaluation of the marginal contribution of genes and other molecular elements in highly interdependent contexts, overcoming several limitations associated with conventional statistical methods. Throughout the manuscript, we discuss the applicability of this method, which demonstrates its capacity to reveal biologically relevant signals, improve the interpretability of models, and enhance analytical reproducibility. Our results demonstrate that the proposed coalitional framework not only enhances the detection of biologically meaningful signals but also improves the interpretability and robustness of omics analyses, particularly in settings characterized by noise, heterogeneity, and cooperative gene effects. Taken together, these advances solidify game theory as a promising tool for integrating highly complex analyses in systems biology and for supporting the development of precision medicine strategies.

## Figures and Tables

**Table 1:** Normalized expression values from a hypothetical gene expression experiment where nine genes were included. C: control samples. E: experimental samples (cases).

	C1	C2	C3	C4	C5	E1	E2	E3	E4	E5
gene1	4.817	4.906	4.942	4.920	4.909	4.770	5.050	4.859	4.752	5.353
gene2	4.771	4.649	4.819	4.706	4.195	4.705	4.574	4.317	4.574	4.480
gene3	3.678	3.610	3.669	3.817	3.714	3.628	3.560	3.624	3.502	3.728
gene4	2.972	3.069	3.214	3.334	3.069	3.022	3.181	3.168	3.021	3.177
gene5	3.470	3.528	3.528	3.719	3.472	3.417	3.514	3.517	3.267	3.706
gene6	4.982	5.110	5.284	5.182	5.759	4.645	5.060	5.196	4.632	5.589
gene7	4.201	3.868	4.048	3.981	3.868	3.903	3.983	3.817	3.778	3.663
gene8	3.326	3.309	3.346	3.492	3.392	3.259	3.285	3.332	3.207	3.400
gene9	6.279	6.489	6.359	6.660	6.875	6.318	6.538	6.661	6.413	6.591

**Table 2:** Boolean matrix $B^{+}$ obtained from data in Table 1. C: control samples. E: experimental samples (cases).

	C1	C2	C3	C4	C5	E1	E2	E3	E4	E5
gene1	0	0	0	0	0	0	1	0	0	1
gene2	0	0	0	0	0	0	0	0	0	0
gene3	0	0	0	1	0	0	0	0	0	0
gene4	0	0	0	1	0	0	0	0	0	0
gene5	0	0	0	1	0	0	0	0	0	1
gene6	0	0	0	0	1	0	0	0	0	1
gene7	1	0	0	0	0	0	0	0	0	0
gene8	0	0	0	1	0	0	0	0	0	0
gene9	0	0	0	0	1	0	0	0	0	0

**Table 3:** Boolean matrix $B^{-}$ obtained from data in Table 1. C: control samples. E: experimental samples (cases).

	C1	C2	C3	C4	C5	E1	E2	E3	E4	E5
gene1	1	0	0	0	0	1	0	0	1	0
gene2	0	0	0	0	1	0	0	1	0	0
gene3	0	1	0	0	0	0	1	0	1	0
gene4	1	0	0	0	0	0	0	0	0	0
gene5	0	0	0	0	0	1	0	0	1	0
gene6	0	0	0	0	0	1	0	0	1	0
gene7	0	0	0	0	0	0	0	1	1	1
gene8	0	0	0	0	0	1	1	0	1	0
gene9	1	0	0	0	0	0	0	0	0	0

**Table 4:** Boolean matrix $B^{E+}$ and the corresponding Shapley values (Sh). Average marginal contribution: meanSh.

	E1	E2	E3	E4	E5	ShE1	ShE2	ShE3	ShE4	ShE5	meanSh
gene1	0	1	0	0	1	0	1	0	0	0.333	0.267
gene2	0	0	0	0	0	0	0	0	0	0	0
gene3	0	0	0	0	0	0	0	0	0	0	0
gene4	0	0	0	0	0	0	0	0	0	0	0
gene5	0	0	0	0	1	0	0	0	0	0.333	0.067
gene6	0	0	0	0	1	0	0	0	0	0.333	0.067
gene7	0	0	0	0	0	0	0	0	0	0	0
gene8	0	0	0	0	0	0	0	0	0	0	0
gene9	0	0	0	0	0	0	0	0	0	0	0

**Table 5:** Boolean matrix $B^{E-}$ and the corresponding Shapley values (Sh). Average marginal contribution: meanSh.

	E1	E2	E3	E4	E5	ShE1	ShE2	ShE3	ShE4	ShE5	meanSh
gene1	1	0	0	1	0	0.25	0	0	0.167	0	0.083
gene2	0	0	1	0	0	0	0	0.5	0	0	0.1
gene3	0	1	0	1	0	0	0.5	0	0.167	0	0.133
gene4	0	0	0	0	0	0	0	0	0	0	0
gene5	1	0	0	1	0	0.25	0	0	0.167	0	0.083
gene6	1	0	0	1	0	0.25	0	0	0.167	0	0.083
gene7	0	0	1	1	1	0	0	0.5	0.167	1	0.333
gene8	1	1	0	1	0	0.25	0.5	0	0.167	0	0.183
gene9	0	0	0	0	0	0	0	0	0	0	0

**Table 6:** Boolean matrix $B^{C+}$ and the corresponding Shapley values (Sh). Average marginal contribution: meanSh.

	C1	C2	C3	C4	C5	ShC1	ShC2	ShC3	ShC4	ShC5	meanSh
gene1	0	0	0	0	0	0	0	0	0	0	0
gene2	0	0	0	0	0	0	0	0	0	0	0
gene3	0	0	0	1	0	0	0	0	0.25	0	0.05
gene4	0	0	0	1	0	0	0	0	0.25	0	0.05
gene5	0	0	0	1	0	0	0	0	0.25	0	0.05
gene6	0	0	0	0	1	0	0	0	0	0.5	0.1
gene7	1	0	0	0	0	1	0	0	0	0	0.2
gene8	0	0	0	1	0	0	0	0	0.25	0	0.05
gene9	0	0	0	0	1	0	0	0	0	0.5	0.1

**Table 7:** Boolean matrix $B^{C-}$ and the corresponding Shapley values (Sh). Average marginal contribution: meanSh.

	C1	C2	C3	C4	C5	ShC1	ShC2	ShC3	ShC4	ShC5	meanSh
gene1	1	0	0	0	0	0.333	0	0	0	0	0.067
gene2	0	0	0	0	1	0	0	0	0	1	0.2
gene3	0	1	0	0	0	0	1	0	0	0	0.2
gene4	1	0	0	0	0	0.333	0	0	0	0	0.067
gene5	0	0	0	0	0	0	0	0	0	0	0
gene6	0	0	0	0	0	0	0	0	0	0	0
gene7	0	0	0	0	0	0	0	0	0	0	0
gene8	0	0	0	0	0	0	0	0	0	0	0
gene9	1	0	0	0	0	0.333	0	0	0	0	0.067

**Table 8:** Absolute difference in the Shapley values (ADSVs) related to $B^{C+}$ and $B^{E+}$ matrices. In bold, ADSVs greater than the mean plus the standard deviation for each group (0.130). CASh: uncorrected *p*-value obtained with the bootstrap resampling procedure. Average marginal contribution: control (meanShC) and experimental (meanShE) groups.

	meanShC	meanShE	ADSV	CASh
gene1	0	0.267	**0.267**	0.1
gene2	0	0	0	1
gene3	0.05	0	0.05	0.34
gene4	0.05	0	0.05	0.34
gene5	0.05	0.067	0.017	0.7
gene6	0.1	0.067	0.033	0.84
gene7	0.2	0	**0.2**	0.37
gene8	0.05	0	0.05	0.34
gene9	0.1	0	0.1	0.22

**Table 9:** Absolute difference in the Shapley values (ADSVs) related to $B^{C-}$ and $B^{E-}$ matrices. In bold, ADSVs greater than the mean plus the standard deviation for each group (0.181) and significant uncorrected *p*-values obtained with the CASh bootstrap resampling procedure. Average marginal contribution: control (meanShC) and experimental (meanShE) groups.

	meanShC	meanShE	ADSV	CASh
gene1	0.067	0.083	0.017	0.78
gene2	0.2	0.1	0.1	0.55
gene3	0.2	0.133	0.067	0.67
gene4	0.067	0	0.067	0.41
gene5	0	0.083	0.083	**0.04**
gene6	0	0.083	0.083	**0.04**
gene7	0	0.333	**0.333**	**0.04**
gene8	0	0.183	**0.183**	**0.02**
gene9	0.067	0	0.067	0.41

## Data Availability

Custom scripts for data analysis using game theory approaches were deposited by our group in the public repository Zenodo and are available through https://zenodo.org/records/11222132 (accessed on 9 December 2025). Additional implementation details and supporting materials are available from the corresponding authors upon request.

## Abbreviations

The following abbreviations are used in this manuscript:

ASD	Autism Spectrum Disorder
CASh	Comparative Analysis of Shapley values
meanSh	Mean Shapley value (Average marginal contribution)
